# Sickness absence, marginality, and medically unexplained physical symptoms: A focus-group study of patients’ experiences

**DOI:** 10.3109/02813432.2013.788274

**Published:** 2013-06

**Authors:** Aase Aamland, Erik L. Werner, Kirsti Malterud

**Affiliations:** ^1^Research Unit for General Practice, Uni Health, Bergen, Norway; ^2^Department of Global Public Health and Primary Care, University of Bergen, Norway

**Keywords:** Focus groups, general practice, medically unexplained physical symptoms, Norway, qualitative research, sick leave

## Abstract

**Purpose:**

Medically unexplained physical symptoms (MUPS) form a major cause of sickness absence. The purpose of this study was to explore factors which may influence further marginalization among patients with MUPS on long-term sickness absence.

**Methods:**

Two focus-group discussions were conducted with a purposive sample of 12 participants, six men and six women, aged 24–59 years. Their average duration of sickness absence was 10.5 months. Participants were invited to share stories about experiences from the process leading to the ongoing sickness absence, with a focus on the causes being medically unexplained. Systematic text condensation was applied for analysis. Inspired by theories of marginalization and coping, the authors searched for knowledge of how patients’ positive resources can be mobilized to counteract processes of marginality.

**Results:**

Analysis revealed how invisible symptoms and lack of objective findings were perceived as an additional burden to the sickness absence itself. Factors that could counteract further marginalization were a supportive social network, positive coping strategies such as keeping to the daily schedule and physical activity, and positive attention and confidence from professionals.

**Conclusions:**

Confidence from both personal and professional contacts is crucial. GPs have an important and appreciated role in this aspect.

- Medically unexplained physical symptoms (MUPS) are a leading cause of sickness absence, but few studies focus on personal consequences from sickness absence.
- The current study adds to this field by exploring factors influencing marginalization among MUPS patients on long-term sickness absence.
- Personal coping strategies such as a strict daily schedule and physical activity may prevent further marginalization.
- The additional burden experienced by these patients due to the invisible symptoms may be eased by support and confidence from family members, friends, and professionals, especially GPs.

## Introduction

Medically unexplained physical symptoms (MUPS) include conditions characterized by subjective symptoms without corresponding objective findings [1–3]. After adequate medical assessment, such symptom presentations may be for example asthenia, chronic fatigue syndrome, fibromyalgia, irritable bowel syndrome, or chronic low back pain [3]. MUPS do not signify psychiatric disorder, although comorbidity often appears [3]. MUPS has been suggested to be a leading cause for sickness absence [4–6].

Sickness absence represents a marginalized state and may challenge personal identity and coping. We understand *marginalization* as a continuum between “included” and “excluded” from working life [7]. MUPS may contribute to further marginalization, since these conditions are attributed low medical status [8]. *Coping* is “thoughts and behaviours used to manage internal and external demands of situations that are appraised as stressful” [9].Theories of marginalization [7] and coping [9] can be used to understand how patients’ positive resources can be mobilized to counteract processes of marginality [10].

There is a lack of studies regarding the personal consequences of sickness absence [5]. As general practitioners (GPs) with various length of clinical experience, we shared an interest in marginalized groups of patients in general, and more specifically patients with MUPS. We therefore wanted to explore experiences from sickness absence in this group of patients, focusing especially on marginalization.

## Design, material, and methods

A focus-group design was chosen to take advantage of the communicative interaction between participants sharing their experiences [11–12]. Participants were recruited from a Norwegian rural health centre with 13 GPs serving a total of 12 063 patients. The GPs recruited patients with the following inclusion criteria: age over 18 years, previous work experience for at least two years, and ongoing sickness certification lasting for 3–12 months due to a diagnosis characterized by the lack of objective findings.

Our sample included six men and six women aged 24–59 years with average duration of sickness absence of 10.5 months. Ten were married or co-habiting. No participants were educated beyond secondary school, and most of them were blue-collar workers. Their diagnoses included fibromyalgia, asthenia, low back pain, and other muscular pain conditions. We organized the focus groups by gender to reduce a potential “peacock effect” [13], since conversations about coping and marginality may be gendered [14]. However, the design did not allow for comparative analysis of possible gender differences. The groups met once for 90 minutes’ discussion.

Participants were invited to share experiences from the processes leading to the ongoing sickness absence, focusing on the fact that their health problems were regarded as medically unexplained. The participants’ experiences of the sickness absence were also requested. Ethical approval for the study was approved by the Regional Committee for Medical and Health Research Ethics.

Audio-recorded interviews were transcribed verbatim by the first author. All authors collaborated on analysis conducted as systematic text condensation [15–16]. The four steps comprised: (i) reading all the material to obtain an overall impression and bracketing previous preconceptions, (ii) identifying units of meaning, representing different aspects of participants’ marginality experiences from sickness absence and coding for these, (iii) condensing the contents of each of the coded groups, and (iv) summarizing the contents of each code group to generalize descriptions and concepts concerning marginality and sickness absence due to MUPS.

## Results

Analysis revealed that invisible symptoms and lack of objective findings were in themselves perceived as an additional burden of sickness absence. Factors that could prevent further marginalization were a supportive social network, positive personal coping strategies such as keeping to the daily schedule and being physically active, and positive attention and confidence from colleagues and professionals. These findings are further elaborated below. Quotations are assigned pseudonyms.

### The invisibility of symptoms increases the burden of sickness absence

There was broad agreement among participants that the lack of objective findings represented a significant additional burden. This was explained by perceived lack of understanding from family, friends, and professionals (employers, doctors, and Norwegian Labour and Welfare Administration officers [NAV]), as many of them called for evidence, such as visible handicap, objective findings, or “real” diagnoses. Several participants stated that they thought a plastered arm or leg, or reference to pathological findings would be more convincing. A couple of participants also described how bad they felt if they were observed at a time when they were expected to be at work. A man in his thirties working in a factory stated:

It hurts to suffer from illness which nobody can see. It really tears [at you]. (Bob)

In different ways, participants described how the lack of proof could be a disadvantage in meetings with NAV. A man who had been on sickness absence several times during his life had initiated a meeting at the local NAV office because he hoped for re- education. However, he was told that his lack of a “real” diagnosis made this impossible. A woman in her fifties with widespread muscular pain described her experience thus:

I think it is easier to do follow-up with more concrete diseases…. Nobody actually knows what is wrong with me, it is so hidden…. I apparently look normal from the outside, but nobody knows how I feel inside…. (Jane)

### Friends and family become especially important when everyday social contact with workmates is reduced

The sickness absence evoked feelings of sorrow and uselessness among several participants, much due to loss of the regular social interactions at work. They described how their absence made them feel like strangers. A man working in a warehouse explained the importance of the social network at his job like this:

That is the most important part of my job, the social. If you are gone for a long while, it changes, [it is] as if you have to rebuild it all up again. (James)

Some participants described how sickness absence had made them more passive and isolated, and they explained the importance of a network to prevent further isolation. Friends and families, especially their spouses, had contributed to decisive support. Some emphasized that they preferred their friends to contact them, because they felt less initiative to do so themselves. A married woman in her fifties said:

… to be sick … you have to adapt yourself … you just have to adapt, isn't that so? For me it has been a lot of downs, but I have pulled myself together, also with help from both my husband and my friends who said: you must not just sit about! (Rose)

### The transition from work life to sickness absence evokes ambiguous emotions

Participants described strong feelings of relief when they finally received the sickness certification, since they had longed for an opportunity to wind down. But for most of them it soon turned into feelings of failure, because they would rather work, feel useful, and contribute. We also heard descriptions of emotional reactions such as irritation, restlessness, or depressive feelings. Approaching a state of reconciliation was described as a tough issue by participants, for some of them a long and ongoing process. A man working as an electrician described his ambiguous feelings regarding his sickness absence as follows:

It was a relief; I suddenly got the opportunity to catch up again. But on the other hand it was really … how should I describe it … like degradation or a humiliation to be on leave. I do not want to be on leave…. (Chris)

A few participants described how they intended to return to work, although they did not feel that their illness had improved. They mentioned feelings of “giving up”, and expressed an expectation of another sickness certification in the near future. A truck driver with shoulder pain planned to return to work because further absence would have economic disadvantage. He still had painful symptoms, but commented:

Yes, I intend to start working again, and time will show how long I will manage this time. We will see.... (Jonathan)

Several participants described how the transition from work life to sickness absence made them reconsider life values and their own feeling of self. A few described how they had gradually developed strategies for protection against prejudices from themselves or from others. Such strategies were an open and direct communication to reduce potential suspicion and uncomfortable questions, or efforts to ignore what others may think of them. The unpredictability of the situation regarding possible outcomes, prognosis, and the process of how to return to work was described as a stressful puzzle.

Yet, the participants described other coping strategies, including a belief that they would manage. A woman said that although she would probably never return to full-time work, at least she had decided to manage her health problems. Some mentioned the importance of maintaining a daily schedule. One woman had seen an opportunity to acquire a hobby or spend more time with her grandchildren. Others talked about positive experiences related to physical activity, like walking in the fields. Activity-group programmes with a low entrance threshold were praised. A woman living in a rural area who was feeling isolated told of how she felt about being invited to an activity group by her doctor:

It is so difficult to get started on your own, I think, so for me the suggestion from the doctor was very good. Getting started. (Mary)

### Positive attention and confidence from workplace and professionals is essential

Some participants described how they maintained contact with the workplace by calling or visiting. For most of them such efforts were positive experiences, although with a few exceptions. Such contact could be obtained through direct meetings with colleagues or the employer, or by telephone. Several participants said that they preferred to be contacted, and described how important it was to be reassured that they were missed by their colleagues. A woman who had remained in the same job for many years admitted:

I would never have shown up at work unless they had called me and encouraged me to come by. If not, I would just have stayed at home. (Kate)

Most of the participants expressed satisfaction with their GPs. The need for time, empathy, and trust was of great importance. Satisfaction could also be enhanced when sufficient medical investigations had been undertaken, either GP-initiated, patient- initiated, or combined. However, several participants called for a more proactive doctor, one who would take initiatives towards examination, treatments, or information about enterprises. A cleaner in the process of being relocated to another type of job described the important role of her doctor like this:

My doctor has been just fantastic. He has listened, so that I have the ability to get my frustration out, and put my problems into words. He has helped me to where I am today. (Jane)

Several participants felt that NAV pushed the person back towards work as fast as possible, independent of their functional abilities. Many participants were frustrated over a system they looked upon as rigid, feeling for instance that their diagnosis was more important to NAV staff than their actual function. A man stated that he felt much worse for several days after a meeting at the NAV office since he had felt pushed in a negative way. Yet, some positive experiences and outcomes were described, attributed to constructive cooperation between NAV, their workplace, and their GPs, such as in Jane's case. A man with asthenia described relief about having limited contact with NAV:

I have not had much contact with NAV. But for me that is really like a blessing! I feel so bad, so if they had pushed me, I think I would have been even worse. (Chris)

## Discussion

The lack of objective findings was perceived as an additional burden. The importance of a social network, as well as different personal coping strategies, was described. Confidence from professionals was crucial. Below, we discuss the strengths and limitations of these findings.

### Methodological challenges

Our sample of participants represented a range of age and actual length and grade of sickness absence. The diagnoses varied. Two to five groups are often recommended when conducting focus-group interviews [17]. There is, however, no consensus on sample size in focus groups, and the main question is whether the data are sufficiently diverse and rigorous to answer the research question [18,19]. After conducting two groups, we critically read the transcripts. We found the material sufficiently abundant for relevant events.

Our aim was to focus participants’ experiences of sickness absence, not to explore their illness trajectories, although ewe were aware of the tight connections between these two issues. During the discussions we therefore focused particularly on the process of being excluded from working life, and found that the participants shared stories representing experiences from this rather than their symptom descriptions.

Nevertheless, talking about marginality is a sensitive issue. We succeeded in establishing a safe atmosphere in the group, enhancing openness and honesty, confirmed by the fact that participants shared quite differing stories. Yet, there is a lot about the participants which we did not assess, for instance negative perpetuating factors. Most of the participants claimed that they wanted to return to work. We have not, however, investigated their actual job involvement, and we do not know whether they finally returned to work or not.

### Sickness absence and marginality

Studies on personal consequences of long-term sickness absence are few and mostly concern adverse financial and social consequences [20–22]. In contrast to these only one participant mentioned financial concern, and none worried about a lack of possible promotion in our study.

Floderus et al. present some positive consequences from sickness absence among women, such as improved sleep and contact with children [21]. Among our participants, only one woman stated enhanced contact with grandchildren as a positive benefit of sickness absence. Previous studies are consistent with the current findings, that sickness absence provides mainly negative consequences, such as feelings of shame and uselessness [21], inactivity, isolation, and depression [21,23].

Furthermore, almost none of our participants called for re-education, in contrast to the findings from a study on sickness absence due to burnout [24]. In contrast to the findings from a study on sickness absence due to musculoskeletal problems [25], none of our participants regarded life at home as more important than their working life. Most of them expressed a strong bond to their work, and almost all of them expressed a clear wish to return to work. We were struck by their overall descriptions of loss, contrasting with a previous study where blue-collar female workers revealed a fragile job identity [26]. These findings diverge from those of Sieurin et al., who described a loss of drive to work at all, even among those actually back at work after a period of sickness absence [22].

The current study adds to existing knowledge by revealing specific consequences of sickness absence for patients with MUPS conditions. Participants presented important messages about how the invisibility of their symptoms imposed a heavy extra burden, adding to previous knowledge about the frustration felt due to lack of proof [27].

A recent study supports our findings and demonstrates the impact of social support from significant others and positive coping strategies in preventing psychological distress because of long-term sickness absence [28]. Also a study from a rehabilitation centre described how a successful return to work was achieved among individuals who were able to control their life situations and manage their jobs despite their complaints [29], and individuals who received a disability pension presented less self-control. The latter externalized their problems to, for instance, lack of competence among professionals, such as lack of follow-up from employers. This resembles our findings in that some participants expressed a wish to be contacted by the employer rather than to call or show up themselves.

The participants in our study seemed to be more satisfied with their GPs than previously reported [23]. Sufficient time, empathy, and confidence were mentioned as important by our participants. A recent literature review regarding treatment strategies for MUPS patients concludes along the same lines emphasizing such traditional general ideals as patient-centred communication, doctor–patient alliances, and regular meetings [30]. Finally, our study elucidates and concretizes the impact of personal relations, both private and professional, during sickness absence [31]. A few participants described how reduced presence at their job due to ongoing illness might have prevented further marginalization, supporting a Swedish study concerning part-time sickness absence [22]. GPs’ encouragement to remain active seems to be important. General factors such as sufficient time, empathy, and confidence alongside a focus on and encouragement of the patient's own positive coping strategies may prevent further marginalization from working life.
